# Redifferentiation therapy with selpercatinib in RET-fusion-positive advanced thyroid carcinoma: a case report and literature review

**DOI:** 10.3389/fonc.2026.1819030

**Published:** 2026-06-29

**Authors:** Edoardo Talpacci, Silvia Morelli, Chiara Ingriccini, Antonella Ferrieri, Cristina Tranfaglia, Massimo Eugenio Dottorini, Efisio Puxeddu

**Affiliations:** 1 Section of Internal Medicine and Endocrine and Metabolic Sciences, Department of Medicine and Surgery, University of Perugia, Perugia, Italy; 2 Department of Diagnostic Imaging, Nuclear Medicine Unit, Perugia General Hospital, Perugia, Italy

**Keywords:** case report, redifferentiation, RET-fusion-positive RAI-R-DTC, review, selpercatinib

## Abstract

Radioiodine-refractory differentiated thyroid carcinoma (RAI-R-DTC) represents a major therapeutic challenge, and strategies aimed at restoring iodine avidity are of increasing clinical interest. We report the case of a 58 year old woman with metastatic papillary thyroid carcinoma harboring a CCDC6:RET fusion, who developed progressive RAI-refractory disease decades after initial surgery and multiple ^131^I-treatments. After a first-line cycle with Lenvatinib characterized by limited efficacy and poor tolerability, a second-line therapy with the selective RET inhibitor Selpercatinib was initiated, resulting in a sustained partial radiological response. Notably, after 17 months of treatment, a diagnostic ^131^I whole-body scan following rhTSH stimulation demonstrated radioiodine uptake restoration in distant metastases, enabling successful retreatment with ^131^I. This was followed by a marked biochemical response and further reduction in tumor burden, with durable disease control under combined therapy. This case illustrates Selpercatinib-induced redifferentiation in RET fusion–positive RAI-R-DTC and supports the emerging role of genotype-driven targeted therapies to restore radioiodine sensitivity. A review of the available literature underscores the potential of selective RET inhibition as a redifferentiation strategy, although further prospective studies are needed to define optimal patient selection and treatment sequencing.

## Introduction

The sodium iodide symporter (NIS), selectively expressed by thyroid follicular cells, has enabled radioiodine therapy to become a cornerstone in the treatment of differentiated thyroid cancer (DTC). It relies on the administration of radioactive iodine (^131^I), either as empirically prescribed activities, typically ranging from 1.1 to 7.4 GBq (30–200 mCi) according to the therapeutic objectives, or following individualized dosimetric assessment (1). Although generally well tolerated, ^131^I-therapy is associated with potential somatic adverse effects, most commonly radiation-induced thyroiditis of thyroid remnants, acute and/or chronic sialadenitis and gastritis, as well as stochastic effects, including the induction of secondary malignancies, leukemia, and impairment of fertility (2–5). To maximize iodine uptake by residual thyroid follicular cells, treatment is preceded by either stimulation with recombinant human thyroid-stimulating hormone (rhTSH) or temporary withdrawal of thyroid hormone replacement therapy (6). ^131^I -therapy may be indicated either for remnant ablation to eradicate potential neoplastic foci within residual thyroid tissue and to facilitate subsequent biochemical and imaging surveillance, or as adjuvant therapy in patients at increased risk of recurrence, with the aim of treating potential microscopic extra-thyroidal metastatic disease. Furthermore, in patients with iodine-avid metastatic DTC, ^131^I -therapy remains an essential treatment and may be administered with curative intent (7).

However, while ^131^I-therapy leads to remission in approximately one third of metastatic DTC, the remaining 60-70% ultimately develop radioiodine-refractory (RAI-R) disease (8). Aberrant MAPK pathway activation largely contributes to radioiodine refractoriness through downregulation of the NIS and thyroid differentiation genes, providing the rationale for investigating selective tyrosine kinase inhibitors (TKIs) as redifferentiating agents (9).

Accordingly, Alan L. Ho et al. were the first to demonstrate the restoration of ^131^I avidity after therapy with MEK1 and MEK2 inhibitor Selumetinib in a subgroup of RAI-R-DTC (10). Since then, several published prospective phase 2 studies and case reports, mainly involving BRAF and MEK inhibitors, have evaluated the potential restoration of ^131^I uptake after MAPK pathway inhibition and the efficacy of additional ^131^I-therapies (11). In detail, administration of ^131^I therapy following TKI-induced redifferentiation aims to maximize TKIs therapeutic efficacy while limiting prolonged TKI exposure, thereby reducing long-term associated toxicities and the risk of acquired resistance.

RET proto-oncogene rearrangements are detected in approximately 10–20% of papillary thyroid carcinomas (PTC) (12). Selpercatinib, a highly selective RET inhibitor, is currently approved for the treatment of previously treated RET fusion–positive RAI-R-DTC (13). However, despite its established therapeutic efficacy, the potential of Selpercatinib to restore radioiodine avidity in RET fusion–positive RAI-R-DTC remains largely unexplored and is limited to a few clinical reports. This report aims to further corroborate the role of Selpercatinib as a redifferentiating agent by contextualizing our findings within the existing literature.

## Case presentation

The patient, a 58-year-old female with unremarkable medical and drug history, presented at our Institution in May 2021 following the finding of thyroid disease progression. The patient underwent total thyroidectomy with central compartment and left latero-cervical lymph nodes dissection in 1985, because of a locally invasive classic variant of PTC involving the left thyroid lobe with synchronous pulmonary micrometastases (pTx,N1b,M1 staging according to Tumor-Node-Metastasis (TNM) classification (14)). Between 1985 and 1989 the patient underwent eight cycles of ^131^I-therapy with a cumulative ^131^I activity of 800 mCi, finally resulting in the disappearance of pulmonary uptake.

### Diagnostic assessments

The following assessments showed biochemical incomplete response (suppressed thyroglobulin values up to 2 ng/ml) in absence of localizable disease until May 2021 when thyroglobulin (Tg) value quickly rose to 50 ng/ml despite a negative diagnostic I^131^ whole-body scan (WBS). The 18FDG-PET/TC scan subsequently performed showed both mediastinal lymph nodes and bilateral pulmonary uptake, indicating a progressive RAI-R-DTC. Before first-line systemic therapy start, EBUS-guided biopsy of a carinal lymph node was performed, revealing metastatic high-grade PTC (PAX8+, TTF1+, thyroglobulin +/-). Subsequent next-generation sequencing (NGS) analysis revealed CCDC6–RET (RET/PTC1) fusion. TC scan identified three target lesions with a cumulative diameter of 54.9 mm, according to RECIST 1.1 (15): a right inferior pulmonary lobe lesion of 10.9x11 mm and two mediastinal lymph-nodes of 21.6x37.6 mm and 22.3x27.7 mm, respectively (**Table 1**).

**Table 1:** Morphological response to Selpercatinib according to RECIST 1.1.

Date	Target 1 (mm)	Target 2 (mm)	Target 3 (mm)	Sum (mm)	BTR (%)
07.09.21	21.6	22.3	11.0	54.9	**-**
26.10.21 Starts Lenvatinib					
29.04.22	17.0	23.1	15.0	55.1	**+0.4 (SD)**
23.06.22 Starts Selpercatinib					
07.06.23	8.5	12.7	7.2	28.4	**-48.5 (PR)**
07.02.24 ^131^I treatment					
27.08.24	7.3	N.V.	6.6	13.9	**-74.7 (PR)**
29.08.25	5.0	N.V.	7.0	12.0	**-78.1 (PR)**

*BTR, Best Tumour Response; SD, Stable Disease; PR, Partial Response.

### Treatment strategies and redifferentiation

Hence, the patient underwent Lenvatinib therapy for 7 months. Deviating from standard practice, the dose was up titrated from 4 to 24 mg due to a metastatic subcarinal lymphnode proximity to the bronchial bifurcation and the uncertain presence of a clear dissection plane between the metastasis and the airway, in order to reduce the risk of fistula formation. The patient achieved, at best, stable disease (SD) according to RECIST 1.1, with a sum of target lesions measuring 55.1 mm and a best morphological response of +0.4%, along with a significant drop in Tg value down to 6.38 ng/ml (**Tables 1**, **2**; **Figure 1**). Unfortunately, she poorly tolerated Lenvatinib therapy because of a grade 2 hypertension and grade 3 diarrhea refractory to supportive measures. The combination of these adverse events, together with the slight increase in target lesions (+0.4%) that discouraged Lenvatinib dose reduction, led to a switch, in June 2022, to the second-line selective RET inhibitor Selpercatinib at the dose of 160 mg twice daily. To date, the patient has well tolerated the treatment, with the development of grade 1 hypertension and grade 2 skin edema which induced us to reduce Selpercatinib dose to 120 mg twice daily. In the whole period of therapy, a progressive impairment of thyroid hormone blood concentrations with a deep reduction of FT3 requiring a LT3 (5 mcg bid) + LT4 (100 mcg qd) combination therapy was observed (**Table 2**).

**Table 2:** Thyroid function parameters and thyroglobulin levels across the different phases of treatment.

Treatment phases	TSH (uUI/ml)	FT4 (ng/dl)	FT3 (pg/ml)	Tg (ng/ml)	Tg-Ab (UI/mL)
Lenvatinib start (26/10/2021)	0.001	1.77	3.57	75.89	<0.1
Selpercatinib start (23/06/2022)	0.001	1.82	3.76	6.38	<0.1
LT3+LT4 therapy start (12/05/2023)	0.001	1.54	2.29	8.38	<0.1
^131^I treatment (07/02/2024)	4.42	0.76	2.29	11.3	<0.1
April 2024 (two months after ^131^I treatment)	0.020	1.70	2.80	2.89	<0.1
August 2025 (last follow-up)	0.010	1.56	3.08	2.40	<0.1

**Figure 1:**
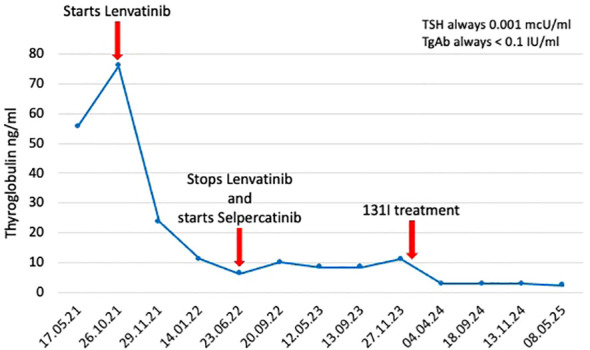
Biochemical response to Selpercatinib and subsequent ^131^I-therapy: timeline of thyroglobulin values.

TC scan performed in June 2023, after one year of therapy with Selpercatinib, showed partial response (PR) according to RECIST 1.1, with a reduction in tumor bulk of 48.5% (at that time the sum of target lesions was 28.4 mm) (**Table 1**). In December 2023, after 17 months of Selpercatinib therapy, a further diagnostic I^131^ WBS after rhTSH was performed showing bilateral pulmonary and mediastinal re-uptake with a stimulated Tg of 39.05 ng/ml​. Of note, one of previously negative pulmonary nodules was smaller and showed intense I^131^ uptake (**Figure 2**). Accordingly, because of the restoration of radioiodine uptake, in February 2024 the patient underwent a further cycle of 147.7 mCi ^131^I-therapy after a 6-week thyroid hormone withdrawal, while Selpercatinib was continued. Notably, despite thyroid hormone withdrawal, the patient remained euthyroid (TSH 4.42 µIU/mL), likely due to hormonally active metastases (**Table 2**). Biochemical assessment after 2 months of therapy (April 2024) confirmed radioiodine efficacy since we could observe a marked reduction in suppressed Tg value, from 11.3 ng/ml before ^131^I-therapy to 2.89 ng/ml after treatment (**Table 2**, **Figure 1**). Furthermore, a CT scan performed in August 2024 confirmed an improvement of the PR, with a reduction in tumor burden from 48.5% to 74.7% (**Table 1**). This response was maintained on a subsequent CT scan in August 2025, showing a further reduction to 78.1% (**Table 1**). In contrast, the ^131^I diagnostic whole-body scan after rhTSH stimulation showed no iodine uptake (stimulated Tg value of 9.9 ng/mL), making the patient ineligible for an additional ^131^I-therapy (**Figure 2**).

**Figure 2:**
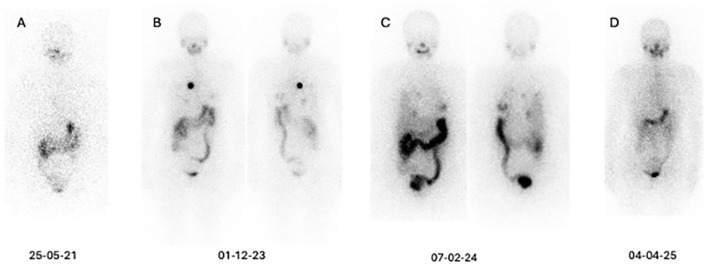
^131^I whole body scans (WBS) performed before and after Selpercatinib therapy. **(A)** ^131^I diagnostic WBS performed on May 2021 (TSH: 76,36 uU/mL; Tg: 413,7 ng/mL), before Lenvatinib initiation, demonstrated no iodine uptake in 18F-FDG avid mediastinal metastases, allowing the definition of RAI-R-DTC. **(B)** ^131^I diagnostic WBS performed on December 2023 (TSH: 189.8 µUI/mL; Tg: 39.05 ng/mL), after 17 months of Selpercatinib therapy, showed bilateral pulmonary re-uptake. **(C)** ^131^I post-therapy (147,7 mCi of ^131^I) WBS (TSH: 4.42 µUI/mL; Tg: 11.3 ng/mL), performed on February 2024, confirmed pulmonary iodine-uptake. **(D)** ^131^I diagnostic WBS performed on April 2025 (TSH: 216,4 uU/mL; Tg: 9,9 ng/mL), 14 months after ^131^I-therapy, showed no uptake.

## Discussion

The present case provides evidence that selective inhibition of RET-driven MAPK pathway signaling may represent a viable strategy to restore iodine avidity. In addition, it highlights biochemical markers supportive of tumor redifferentiation. These findings are particularly relevant because, in contrast to BRAF^V600E^ mutated RAI-R-DTC treated with selective BRAF and MEK inhibitors, clinical evidence supporting redifferentiation induced by selective RET inhibitors remains limited and currently restricted to a small number of case reports. In detail, a comprehensive literature search conducted in the PubMed database identified seven documented cases of RET fusion-positive RAI-R-DTC demonstrating restoration of radioiodine uptake followed by subsequent ^131^I therapy after Selpercatinib treatment (**Table 3**) (16–22).

**Table 3:** Published case reports of Selpercatinib-induced tumor redifferentiation.

Author	Year of publication	Number of patients	RET fusion	Selpercatinib dose	Time to redifferentiation	Iodine avidity restoration based on	Tg before ^131^I therapy	^131^I therapy	Tg after ^131^I therapy
*Groussin L*, et al.	2021	1	NCOA4-RET	160 mg bid	3 weeks	^131^I scan	Basal Tg of 253 ng/ml (before Selpercatinib)	100 mCi (3700 MBq)	Basal Tg of 441 ng/ml
*Lee YA*, et al.	2021	1	CCDC6-RET	80 mg bid	5 months	^123^I scan	Stimulated Tg of 5990 ng/mL (before Selpercatinib)	1°after 5 months-60 mCi (0.11GBq/kg) 2°after 19 months-60 mCi (0.11 GBq/kg)	Stimulated Tg after 1° RAI- 1930 ng/mL Stimulated Tg after 2° RAI- 855 ng/mL
*Werner RA*, et al.	2022	1	RET fusion positive (*not specified*)	*not specified*	15.5 months	^131^I scan	Basal Tg of 148 ng/ml	1°after 15.5 months-9.4 GBq 2°after 20.5 months-7.5 GBq	Basal Tg of 21 ng/ml
*Weiler D*, et al.	2024	1	RET fusion positive (*not specified*)	160 mg bid	9 months	*_*	Basal Tg of 6.76 ng/ml	6672 MBq	Basal Tg of 2.48 ng/ml
*Chiu HK*, et al.	2024	1	RET fusion positive (*not specified*)	120 mg bid	9 months	_	Basal Tg of 5.7 ng/ml	162 mCi (103.5 mCi/m2, 3.1 mCi/kg)	Basal Tg of 0.8 ng/mL
*Barbry F*, et al.	2025	1	NCOA4-RET	*not specified*	6 weeks	_	Basal 6.94 ng/mL (before Selpercatinib)	3.7 GBq	Undetectable
*Tarasova V D*, et al.	2025	1	NCOA4-RET	160 mg bid	3 months	^123^I scan	Basal 79.8 ng/mL	146 mCi (5402 MBq)	Basal Tg of 14.7 ng/mL

Furthermore, prospective data from an ongoing phase II clinical trial are still awaited (NCT05668962).

Redifferentiation of RAI-R-DTC is defined as the restoration of iodine avidity with selective TKI systemic therapy. It implies the expression of thyroid differentiation genes and allows the administration of additional cycles of ^131^I-therapy with curative intent. Across studies, ^124^I PET–CT dosimetry, ^131^I or ^123^I WBS have been used to evaluate iodine uptake. In our case, a ^131^I WBS performed after 17 months of Selpercatinib therapy demonstrated a significant restoration of iodine uptake, with newly evident radioiodine avidity in pulmonary and mediastinal metastases that were previously I^131^ WBS-negative and 18F-FDG PET/CT–positive. Although ^131^I re-uptake in previously non–iodine-avid metastases represents the conventional diagnostic hallmark of tumor redifferentiation, we identified two additional biochemical markers likely supporting this process: 1) the rise in Tg value (11.3 ng/ml vs 6.38 ng/ml) after Selpercatinib therapy initiation despite a marked reduction in tumor burden (-48.5% on the last CT scan prior to ^131^I therapy); 2) the persistence of euthyroidism after replacement therapy withdrawal for ^131^I-therapy administration.

Groussin et al. were the first to report restoration of ^131^I uptake after three weeks of Selpercatinib therapy in a patient with NCOA4–RET–rearranged RAI-R-DTC, allowing subsequent ^131^I treatment. The authors interpreted the observed increase in suppressed Tg levels (253 μg/mL before Selpercatinib vs 441 μg/mL after ^131^I therapy) as a consequence of the redifferentiation process (16). In line with this observation, several published studies have reported a rise in Tg levels in the absence of radiological disease progression during treatment with BRAF or MEK inhibitors (10, 23). Moreover, Jaber et al. described an increase in Tg or anti-Tg antibodies during BRAF or MEK inhibitor therapy in patients who subsequently became eligible for ^131^I therapy due to the restored RAI avidity (24). Similarly, Dunn et al. observed significantly higher Tg levels in ^124^I responders compared with non-responders in a cohort of BRAF-mutant RAI-R-DTC patients treated with Vemurafenib (25).

Similarly to Tg rise, MAPK pathway inhibitors-induced hyperthyroidism or improperly low TSH after replacement therapy discontinuation has been described as a marker of redifferentiation. Specifically, Leboulleux et al. described both a rise in Tg value and the development of clinical hyperthyroidism with increase in FT4 and FT3 (6.6 and 4.4 times their upper reference limit, respectively) after 6-week Dabrafenib and Trametinib therapy in a BRAF^K601E^ mutated thyroid cancer (26). Likewise, Iravani et al. described an inappropriate low increase in TSH value (up to 25 uUI/ml) four weeks after discontinuation of replacement therapy in a patient undergoing redifferentiation treatment (27). Consistently, our patient exhibited an inappropriately low TSH level (4.42 µIU/mL) following replacement therapy withdrawal for ^131^I therapy. In the context of a reduced tumor burden (sum of target lesions 28.4 mm; **Table 1**), this finding was interpreted as a state of relative hyperthyroidism due to hyperfunctioning metastases, which in turn obviated the need for recombinant human TSH stimulation prior to ^131^I administration.

The timing of redifferentiation therapy remains a matter of debate. In our case, restoration of iodine avidity was observed after 17 months. However, timing of Selpercatinib-induced redifferentiation appears to vary considerably across published reports, ranging from 3 weeks, as described by Groussin et al. (16), to 15.5 months, as reported by Werner et al. (22). Clinical evidence remains too limited to define a standardized timeframe for redifferentiation, and these discrepancies may, at least in part, be explained by the well-documented long-term efficacy and tolerability of Selpercatinib (28). Accordingly, in some reports, assessment of radioiodine uptake and subsequent ¹³¹I therapy could have been postponed to allow maximal Selpercatinib-induced reduction of tumor burden and maximize the efficacy of ¹³¹I.

Furthermore, Tarasova et al. reported a sustained biochemical and structural response after ^131^I-therapy, despite subsequent Selpercatinib discontinuation, supporting the persistence of Selpercatinib-induced redifferentiation and the lasting efficacy of ¹³¹I therapy even after drug withdrawal (20).

While the magnitude of the morphological response (−78% vs −48% after I-131 therapy) observed in our case cannot be exclusively attributed to ^131^I administration due to the ongoing Selpercatinib treatment, the substantial decline in serum non stimulated Tg levels just 2 months after ^131^I therapy (2.89 ng/mL vs 11.3 ng/mL) could represent a biochemical indicator of ^131^I efficacy. A decline in serum Tg levels following ¹³¹I therapy represents the most commonly reported finding across published cases of Selpercatinib-induced redifferentiation (**Table 3**). Groussin et al. were the only authors to report an increase in Tg levels; however, as already said, in the context of a marked reduction in tumor burden this finding was interpreted as a surrogate marker of tumor redifferentiation.

Finally, another aspect to be considered regarding the potential redifferentiating efficacy of Selpercatinib is the high prevalence of RET gene rearrangements in pediatric population (29). Lee et al. reported the case of a 7.4-year-old CCDC6:RET fusion–positive metastatic RAI-R-DTC treated with Selpercatinib after locoregional recurrence and lung metastases progression, in which Selpercatinib-induced restoration of radioiodine uptake enambled for two additional ^131^I-treatments with a favorable biochemical response and structural stability (19). Although Selpercatinib therapy was continued in the previous case, the achievement of tumor redifferentiation constitutes a particularly promising clinical opportunity in the pediatric setting. This effect may translate not only into improved disease control but also into the potential to reduce or eventually discontinue systemic therapy. Given their long life expectancy, children and adolescents are especially vulnerable to the cumulative long-term toxicities associated with chronic treatments, making treatment strategies that enable de-escalation particularly valuable for preserving long-term quality of life.

## Conclusion

Like other MAPK pathway inhibitors, Selpercatinib demonstrates potential redifferentiation activity in RET-mutated RAI-R-DTC. Its sustained efficacy and favorable tolerability highlight the importance of identifying the optimal timing for assessing redifferentiation and planning subsequent radiometabolic therapy, as part of a combination strategy that could, in selected cases, allow temporary or permanent discontinuation of systemic treatment. Nevertheless, current evidence remains largely anecdotal, and dedicated clinical trials are needed to evaluate the feasibility of routine use in clinical practice and to define the most appropriate treatment protocols.

## Data Availability

The original contributions presented in the study are included in the article/supplementary material. Further inquiries can be directed to the corresponding author.
